# IVF/ICSI outcomes of euthyroid infertile women with thyroid autoimmunity: does treatment with aspirin plus prednisone matter?

**DOI:** 10.1186/s12884-022-04532-2

**Published:** 2022-03-29

**Authors:** Ping Zhou, Qiuping Yao, Qiaohang Zhao, Lihua Yang, Ya Yu, Jilai Xie, Chun Feng, Liming Zhou, Min Jin

**Affiliations:** 1 Second Affiliated Hospital, School of Medicine, Zhejiang University, 310052 Hangzhou, Zhejiang Province P.R. China; 2 Jiaxing Maternity and Child Health Care Hospital, Zhejiang Province 314051 Jiaxing, P.R. China; 3 Jinhua People’s Hospital, 321000 Jinhua, Zhejiang Province P.R. China; 4 Ningbo Women and Children’s Hospital, 315000 Ningbo, Zhejiang Province P.R. China

**Keywords:** Thyroiditis, Autoimmune, Aspirin, Prednisolone, fertilization*in vitro*

## Abstract

**Background:**

Thyroid autoimmunity (TAI) has been demonstrated to be associated with adverse pregnancy including recurrent miscarriage, unexplained infertility, and implantation failure. To settle with the fertility problem, prescribing aspirin combined with prednisone (P + A) to women positive for anti-thyroid antibodies is frequent in clinical practice, but the underlying effect remains controversial.

**Methods:**

A multicenter, retrospective study was conducted in three reproductive centers from 2017 to 2020. A total of 494 euthyroid infertile women were recruited who were positive for anti-thyroperoxidase and/or thyroglobulin antibodies (TPOAb and TgAb, respectively) with thyroid-stimulating hormone (TSH) levels ranging 0.35-4.0mIU/L and underwent their first *in vitro* fertilization and embryo transfer (IVF-ET) cycle. Ultimately, 346 women were included of which 150 women were treated with prednisone (10 mg/d) and aspirin (100 mg/d). The remaining 196 women were untreated (control group). Treatment started on the day of embryo transfer and continued until clinical pregnancy was determined.

**Results:**

The clinical pregnancy rate was 57.5% vs. 63.5% in the control and treated groups (*P* = 0.414) for first fresh embryo transfer cycles and 57.8% vs. 61.8% for frozen-thawed embryo transfer cycles (*P* = 0.606). In addition, the live birth rate for the fresh embryo transfer was 49.6% vs. 47.3% in the control and treated groups (*P* = 0.762). Logistic regression revealed that aspirin plus prednisone did not improve the clinical pregnancy rate or miscarriage rate. Furthermore, it was observed that low free triiodothyronine (FT3) was associated with high miscarriage rates.

**Conclusions:**

Utilizing an adjuvant treatment of P + A after the embryo transfer may not be necessary in euthyroid women with thyroid autoimmunity undergoing their first IVF-ET, regardless of the embryo type (fresh or frozen).

**Supplementary Information:**

The online version contains supplementary material available at 10.1186/s12884-022-04532-2.

## Background

Despite significant advances in assisted reproductive technology (ART), including controlled ovarian stimulation, assisted hatching, and pre-implantation genetic testing, implantation remains a long-standing rate-limiting step in *in vitro* fertilization (IVF) treatments [1]. Successful implantation is dependent on the intricate collaboration between good-quality embryos and a receptive human endometrium, both of which are indispensable requisites [2–4]. Therefore, when good-quality embryos or even euploid embryos are prepared for the transfer, the endometrium may be responsible for implantation failure [4, 5]. The major factors determining uterine receptivity for implantation and further embryo development are progesterone, estrogens, and the immune system [6]. In addition, the process of reprogramming the maternal immune system from rejection to temporary tolerance towards the fetal (paternally derived) semi-allograft depends on the endocrine-immune interaction [7–9]. Generally speaking, the immune system of a healthy female induces tolerance towards the embryo, whereas this process fails in a hyperactive immune system, thereby reducing fertility and increasing the risk of miscarriage [7]. Among the various studies investigating immunological mechanisms, thyroid autoimmunity, as a predictor of generalized autoimmune disturbance, has been closely linked to recurrent embryo implantation failure, early pregnancy loss, and adverse pregnancy outcomes [10–12]. Furthermore, several reports demonstrated that anti-thyroid antibodies did not affect embryo quality but decreased the clinical pregnancy rate, partly because of the impaired maternal immune modulation [13]. When addressing the reproductive challenges faced by the infertile women who are positive for anti-thyroid antibody pursuing pregnancy, prednisone (P) for immunosuppression and aspirin (A) as an antithrombotic agent are frequently and customarily prescribed in clinical practice.

As a therapeutic alternative, corticosteroid hormones in combination with aspirin may potentially benefit blood perfusion to the ovaries and endometrium and decrease local inflammatory reactions to the transfer procedure, thereby inducing a more favorable microenvironment for the transferred embryo [14, 15]. Furthermore, previous investigations have indicated that combined treatment of P + A is effective for women with various autoimmune diseases [16–19]. However, these trials were published long ago and do not demonstrate the efficacy of this approach in infertile women who are positive only for antithyroid antibodies. Therefore, insufficient evidence exists to determine whether P + A therapy improves the likelihood of a successful pregnancy following ART in thyroid Ab-positive euthyroid women. In addition, some clinicians are more inclined to prescribe combined supplements, even for the first embryo transfer, while others are not.

Hence, the present study aimed to evaluate the effects of aspirin plus prednisone treatment on improving pregnancy outcomes of the first embryo transfer cycle in euthyroid infertile women who only present with positive thyroid autoimmune antibodies.

## Methods

### Patients

In this multicenter retrospective study, a total of 5427 infertile women were included who underwent their first IVF-ET at one of three IVF centers, including the Second Affiliated Hospital of Zhejiang University School of Medicine, Ningbo Women and Children’s Hospital, and People’s Hospital of Jinhua from October 2017 to July 2020. Among them, 597 women were tested positive for TPOAb and/or TgAb. Inclusion criteria were as follows: age under 40 years old, regular spontaneous menstrual cycle (21–35 days), presence of both ovaries, normal ovarian reserve as defined by basic follicle-stimulating hormone (FSH)<10 IU/L, and antral follicle count (AFC)>5.

In addition, infertile patients with thyroid-stimulating hormone (TSH) levels ranging between 0.35 and 4.0 mIU/L, receiving nothing or combined P + A, and undergoing their first *in vitro* fertilization and embryo transfer (IVF-ET) cycle were included in the study. Therefore, a total of 494 infertile women were recruited and analyzed in detail.

Excluded from this study were women with other known autoimmune diseases or clinical presentations of autoimmune disorders including systemic lupus erythematosus (SLE), antiphospholipid syndrome (APS) (*n* = 2), those whose thyroid function was abnormal (*n* = 38), those who were diagnosed with diseases affecting the uterine cavity (*n* = 12), and those whose infertility was caused by severe oligoasthenospermia and azoospermatism (*n* = 16). Similarly, eight women were excluded because they or their partners presented aberrant chromosome karyotypes with particularly significant parental balanced translocations or Turner mosaicism. One woman was excluded because of medical history of insulin-dependent diabetes mellitus (DM), seven women were excluded because of a lack of mature oocytes to retrieve, and 64 women were excluded because of not undergoing any embryo transfer for various reasons.

**Fig. 1:**
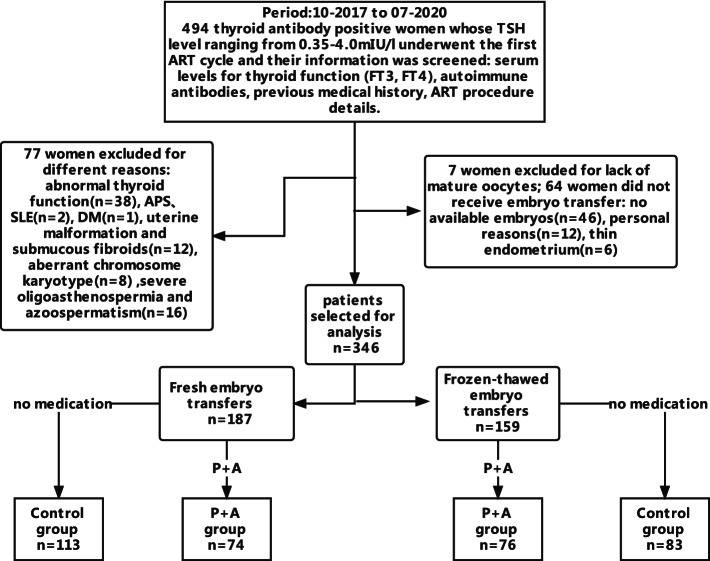
- Flowchart illustrating the selection of the infertile women and their grouping

Of the selected 346 thyroid antibody-positive women, 150 (43.4%) patients received P + A treatment. (Fig. 1). The study was reviewed and approved by the Ethics Committees of the Second Affiliated Hospital of Zhejiang University School of Medicine, Ningbo Women and Children’s Hospital, and People’s Hospital of Jinhua. Based on the trait of a retrospective study, the use of existing medical data, and no risk to patients’ physiology, informed consent was waived by the Ethics Committee.

### Laboratory assays

Serum samples of women were analyzed by the standard third-generation electrochemiluminescence (ECL) immunoassay (CobasElesys 601, Roche) in the three reproductive centers. Thyroid autoimmunity (TAI) was defined as the presence of serum antibodies directed against TPO and/or TG. The reference range was 0–5.61 IU/mL for TPOAb and <4.11 IU/mL for TgAb. Women were diagnosed with euthyroidism when serum TSH levels were within the reference range of 0.35–4.0mIU/L and none of the free thyroxine parameters (FT4) or FT3 was outside the reference values, which were 0.7-1.48ng/dL for FT4 and 1.71-3.71pg/mL for FT3. Reference values were provided by the manufacturer of the assay kits.

### ART procedure and collection of clinical information

In all groups, ovarian stimulation was performed using the following sequential regimen. Firstly, pituitary inhibition was achieved using a gonadotropin-releasing hormone (GnRH) analog (Decapeptyl; Ferring, Switzerland) or GnRH antagonist (Centrotide; Serono, Germany). Secondly, patients underwent ovulation induction with recombinant follicle-stimulating hormone (FSH) (Gonal F; Serono, Switzerland) or human menopausal gonadotropin (HMG) (Livzon, China) to obtain a cohort of mature oocytes at the time of oocyte retrieval. Furthermore, the doses of these drugs were adjusted according to the women’s age, AFC, and day3 FSH values. During that period time, cycles were monitored using transvaginal sonography together with laboratory assays. When a minimum of three leading follicles reached 17–18 mm, paired with appropriate serum E2 levels, a dose of 5000–10,000 IU of human chorionic gonadotropin (hCG) (Livzon, China) was administered. Then Cumulus oocyte complexes were aspirated 36–38 h after hCG injection. Subsequently, whether conventional IVF or ICSI was performed was dependent on the semen condition and clinical indication. All patients were transferred good-quality embryos. Luteal phase support was added in the form of micronized progesterone capsules and oral dydrogesterone. Fourteen days after embryo transfer, serum hCG was assessed and clinical pregnancy was determined 5 weeks after embryo transfer by ultrasonography. Clinical data, including the women’s age, body mass index (BMI), duration of infertility, previous history of miscarriage, FSH, anti-Mullerian hormone (AMH), and AFC were recorded and analyzed. In addition, the following laboratory parameters and pregnancy outcomes were documented: total gonadotropin (Gn) doses, days of gonadotropin treatment, E2 levels on hCG day, endometrial thicknesses on hCG day and embryo transfer day, oocytes retrieved, fertilization rate, number of embryos for transferring, implantation rate of cleavage and blastocyst-stage embryos, pregnancy rate, clinical pregnancy rate, miscarriage rate and live birth rate.

### Adjuvant medical treatments

A total of 346 euthyroid infertile women with thyroid autoimmunity were divided into two groups—the control group (*n* = 196) and the treated group (*n* = 150). Treatment involved orally administered prednisone (Xianju pharmaceutical factory, China) and aspirin (Bayer, Germany) in a daily dose of 10 mg prednisone and 100 mg aspirin. Medication was given starting on the day of embryo transfer and continued until a successful clinical pregnancy was determined by ultrasound. Meanwhile, medication was discontinued if a persistent decline in hCG value occurred.

### Outcome measures

The primary outcome was a clinical pregnancy rate after the first embryo transfer. Secondary outcomes included implantation rate of cleavage-stage embryos, miscarriage rate, and live birth rate after the first embryo transfer.

The pregnancy rate was defined as the percentage of transfers with positive serum levels of hCG (≥5 mIU/mL), whereas a clinical pregnancy was defined as the existence of a viable embryo within an intrauterine gestational sac. The spontaneous abortion rate was defined as the ratio between the number of pregnancy losses after sonographic visualization of an intrauterine gestational sac and the number of clinical pregnancies. Recurrent miscarriage was determined by the loss of two or more clinical pregnancies. The implantation rate was calculated as the number of sacs with a fetal heartbeat divided by the total number of embryos transferred. A live birth rate was defined as the percentage of transfers resulting in a live birth.

### Statistics

Data analysis was performed using the Statistics Package for Social Sciences (SPSS 24.0). First, a Kolmogorov-Smirnov test was applied to both groups and variables to evaluate whether the distribution was symmetrical. Continuous data were expressed as the median (25th–75th) when not normally distributed, and as the mean±SD for normally distributed data. Categorical data were calculated as the number (percentage) of cases. Comparisons of quantitative data were analyzed using the Mann–Whitney U test or independent T test and Chi-square or two-sided Fisher’s exact test in case of categorical data.

For the logistic regression analysis, the independent variables were age and FT3 levels in the whole range. Clinical pregnancy rate and miscarriage rate were considered dependent outcomes.

The significance level of alpha was defined at 0.05, and a value of *p* < 0.05 was considered statistically significant.

## Results

### Clinical characteristics

The characteristics of women with thyroid antibodies(+) are shown in Table 1. The clinical descriptive characteristics were broadly comparable between the P + A and non-treated groups and consisted of age, BMI, number of previous miscarriages, duration of infertility, the proportion of primary infertility, FSH, AMH, AFC, TSH, FT4, the ratio of only TPOAb positivity, only TgAb positivity, or TPOAb and TgAb positivity. Furthermore, the cause of infertility was comparable between groups (Supplemental Table SI). The value of FT3 in the P + A treated group was 2.90 ± 0.39pg/mL, which was significantly lower than that in the group of thyroid antibodies-positive untreated subjects (3.05 ± 0.44, *P* = 0.017).

**Table 1:** Characteristics of women with positive antithyroid antibodies

Cycles	Fresh embryo transfer cycles			Frozen-thawed embryo transfer cycles		
	Control group *n* = 113	P + A group *n* = 74	*P* value	Control group *n* = 83	P + A group *n* = 76	*P* value
Age (yrs.)	31.0 (29.0-35.0)	31.5 (29.0-35.0)	0.832	30.0 (28.0-34.0)	30.0 (28.0-34.8)	0.737
<31	44 (38.9%)	26 (35.1%)	0.829	44 (53.0%)	39 (51.3%)	0.727
31-37	63 (55.8%)	43 (58.1%)		35 (42.2%)	31 (40.8%)	
>37	6 (5.3%)	5 (6.8%)		4 (4.8%)	6 (7.9%)	
BMI (kg/m^2^)	22.0 (20.0-24.0)	22.3 (20.2-25.1)	0.336	22.38 ± 2.97	22.09 ± 3.26	0.557
BMI≥25 (kg/m^2^)	21 (18.6%)	21 (28.4%)	0.117	16 (19.3%)	14 (18.4%)	0.890
Previous miscarriages	0.0 (0.0-1.0)	1.0 (0.0-1.0)	0.636	0.0 (0.0-1.0)	0.0 (0.0-1.0)	0.752
≥2	21 (18.6%)	15 (20.3%)	0.775	7 (8.4%)	10 (13.2%)	0.336
Duration of infertility (yrs.)	3.0 (2.0-5.5)	3.0 (1.0-5.0)	0.961	3.0 (1.3-4.0)	2.0 (2.0-4.0)	0.973
<3	53 (46.9%)	32 (43.2%)	0.623	39 (47.0%)	40 (52.6%)	0.477
≥3	60 (53.1%)	42 (56.8%)		44 (53.0%)	36 (47.4%)	
Primary infertility	50 (44.2%)	30 (40.5%)	0.616	42 (50.6%)	35 (46.1%)	0.566
Basal FSH (IU/L)	5.74 ± 1.57	5.67 ± 1.36	0.732	6.2 (4.7-7.1)	5.2 (4.4-6.3)	0.094
AMH (ng/mL)	2.7 (1.7-3.7)	2.9 (1.9-4.2)	0.429	4.3 (2.4-6.5)	3.9 (1.9-6.0)	0.327
AFC	11.0 (7.0-18.0)	14 (10.0-17.0)	0.103	12.0 (9.0-18.0)	14.0 (8.0-17.0)	0.882
TSH (mIU/L)	2.2 (1.5-2.8)	2.0 (1.4-2.7)	0.422	2.22 ± 0.90	1.99 ± 0.86	0.109
FT4 (ng/dL)	1.00 ± 0.14	1.01 ± 0.09	0.475	1.0 (0.9-1.1)	1.0 (1.0-1.1)	0.867
FT3 (pg/mL)	3.05 ± 0.44	2.90 ± 0.39	0.017^a^	2.99 ± 0.48	2.89 ± 0.39	0.145
Only TPOAb positivity	9 (8.0%)	7 (9.5%)	0.542	9 (10.8%)	12 (15.8%)	0.592
Only TgAb positivity	52 (46.0%)	28 (37.8%)		33 (39.8%)	31 (40.8%)	
Both positivity	52 (46.0%)	39 (52.7%)		41 (49.4%)	33 (43.4%)	

*P* < 0.05 was considered statistically significant. ^a^ represents a statistically significant difference between the two groups

Continuous data are expressed as mean±SD when normally distributed, or as median (25th -75th ) otherwise. Categorical variables are expressed as number and percentage

*BMI* body mass index, *FSH *follicle-stimulating hormone, *AMH *anti-Mullerian hormone, *AFC *antral follicle count, *TSH *thyroid-stimulating hormone, *FT4* free thyroxine, *FT3 *free triiodothyronine, *TPOAb *anti-thyroperoxidase antibody, *TgAb *thyroglobulin antibody

### Cycle characteristics and embryological data

No significant differences were observed in the ratio of GnRHant/GnRHa, days of ovarian stimulation, total Gn doses, E2 levels on hCG day, endometrial thicknesses on hCG day, number of oocytes retrieved, or the type of ART used between the two groups for both fresh and frozen embryo transfer cycles (Table 2).

**Table 2:** Cycle characteristics and embryological data of studied groups

Cycles	Fresh embryo transfer cycles			Frozen-thawed embryo transfer cycles		
	Control group *n* = 113	P + A group *n* = 74	*P* value	Control group *n *= 83	P + A group *n* = 76	*P* value
GnRHant/GnRHa Total Gn Dose (IU)	28/85 2250.0 (1575.0-2700.0)	23/51 2250.0 (1762.5-2925.0)	0.344 0.373	38/45 1950.0 (1425.0-2550.0)	27/49 1875.0 (1425.0-2225.0)	0.189 0.437
Stimulation length (d)	10.0 (9.0-11.0)	10.0 (9.0-11.0)	0.442	9.0 (8.0-11.0)	9.0 (8.0-11.0)	0.897
E2 level on HCG day (pg/mL)	2246.77 ± 980.18	2287.10 ± 865.47	0.547	3449.1 (2076.4-4496.1)	3811.1 (2293.9-5959.9)	0.486
Endometrial thickness on hCG day (mm)	12 (10-12)	12 (10-12)	0.444	/	/	/
Number of oocytes retrieved	9.0 (5.0-12.0)	10.0 (6.0-15.0)	0.053	12.0 (9.0-17.0)	15.5 (9.0-21.8)	0.057
IVF/ICSI	102/11	70/4	0.287	70/13	65/11	0.834
Fertilization rate (%)	82.6 (846/1024)	80.4 (634/789)	0.217	81.0 (856/1057)	78.0 (973/1247)	0.080
Cleavage rate (%)	92.7 (784/846)	93.5 (593/634)	0.519	95.7 (819/856)	94.3 (918/973)	0.194
Available embryo rate (%)	61.2 (480/784)	56.2 (333/593)	0.058	56.8 (465/819)	55.4 (509/918)	0.577

*P* < 0.05 was considered statistically significant

Continuous data are expressed as mean±SD when normally distributed, or as median (25th-75th) otherwise. Categorical variables are expressed as number and percentage

*GnRHant *gonadotropin-releasing hormone antagonist, *GnRHa *gonadotropin-releasing hormone agonist, *Gn dose *gonadotropin dose, *E2 *estradiol, *hCG* human chorionic gonadotropin, *IVF* *in vitro* fertilization, *ICSI *intracytoplasmic sperm injection

### Reproductive outcomes

As for fresh embryo transfers, outcomes were observed and documented (Table 3). The implantation rate of cleavage-stage embryos was slightly higher (non-significant difference) in treated women than in untreated women (44.7% vs. 40.2%, respectively; *P* = 0.407). As for clinical pregnancy rate, a higher but non-significant prevalence of clinical pregnancy was observed in treated patients (63.5% vs. 57.5%; *p* = 0.414). More miscarriages were reported in the treated group than in the control group at the first attempt (25.5% versus 13.8%), but this difference was not significant (*P* = 0.118). The prevalence of live births among treated women was 47.3% compared with 49.6% among untreated women (*P* = 0.762). The results regarding frozen embryo transfers were comparable to those obtained with fresh embryo transfers. Notably, an increased but nonsignificant prevalence of cleavage-stage embryo implantation and clinical pregnancy was observed in treated women compared with untreated women (45.5% vs. 39.7%, *P* = 0.341; 61.8% vs. 57.8%, *P* = 0.606). Additionally, the incidence of treated women that suffered a miscarriage was 27.7% vs. 18.8% in the control group (*P* = 0.303). At the first frozen embryo transfer, the likelihood of delivering a live birth was similar between the treated group and the control group (44.7% vs. 47.0%, *P* = 0.776).

**Table 3:** Reproductive outcomes at the first embryo transfer

Cycles	Fresh embryo transfer cycles			Frozen-thawed embryo transfer cycles		
	Control group *n* = 113	P + A group *n* = 74	*P* value	Control group *n* = 83	P + A group *n* = 76	*P* value
Embryo stage, n (%) Cleavage stage	108 (95.6%)	72 (97.3%)	0.544	73 (88.0%)	71 (93.4%)	0.239
Blastocyst stage	5 (4.4%)	2 (2.7%)		10 (12.0%)	5 (6.6%)	
Number of embryos transferred	1.88 ± 0.33	1.93 ± 0.25	0.214	1.81 ± 0.40	1.84 ± 0.37	0.565
Endometrial thickness on embryo transfer day (mm)	/	/	/	9.1 (8.5,11.0)	9.1 (8.5,11.0)	0.984
Implantation rate (%)	40.1 (85/212)	44.8 (64/143)	0.355	42.0 (63/150)	46.4 (65/140)	0.448
Implantation rate of cleavage-stage embryos (%)	40.2 (82/204)	44.7 (63/141)	0.407	39.7 (54/136)	45.5 (60/132)	0.341
Implantation rate of blastocyst-stage embryos (%)	37.5 (3/8)	50.0 (1/2)	/	60.0 (9/14)	62.5 (5/8)	/
Pregnancy rate (%)	63.7 (72/113)	64.9 (48/74)	0.873	65.1 (54/83)	65.8 (50/76)	0.923
Clinical pregnancy rate (%)	57.5 (65/113)	63.5 (47/74)	0.414	57.8 (48/83)	61.8 (47/76)	0.606
Miscarriage rate (%)	13.8 (9/65)	25.5 (12/47)	0.118	18.8 (9/48)	27.7 (13/47)	0.303
Neonatal mortality rate (%)	0 (0/113)	0 (0/74)		0 (0/83)	0 (0/76)	
Live birth rate (%)	49.6 (56/113)	47.3 (35/74)	0.762	47.0 (39/83)	44.7 (34/76)	0.776

*P* < 0.05 was considered to be statistically significant

Continuous data are expressed as mean±SD when normally distributed, or as median (25th-75th) otherwise. Categorical variables are expressed as number and percentage

### Logistic regression analysis

Since there was a significant difference in FT3 at the fresh embryo transfer cycle between women with or without P + A treatment, multiple logistic regression analysis was performed. Moreover, age, the clinically relevant variable, was included in the regression analysis (Table 4). In women who received a fresh embryo, after adjusting for age and additional treatments, FT3 within the normal reference appeared to have a negative relationship with the miscarriage rate (odds ratio [OR] 0.248 [95% confidence interval, CI 0.063-0.984], *P* = 0.047). Furthermore, P + A treatment had no influence on the miscarriage rate or clinical pregnancy rate.

**Table 4:** Multivariable logistic regression analysis

Dependent outcomes	Fresh embryo transfer cycles				Frozen-thawed embryo transfer cycles			
	Miscarriage rate		Clinical pregnancy rate		Miscarriage rate		Clinical pregnancy rate	
Independent variables	aOR [CI]	*P*	aOR [CI]	*P*	aOR [CI]	*P*	aOR [CI]	*P*
Age (yrs.)	0.983[0.850-1.135]	0.812	1.040[0.957-1.130]	0.352	1.002[0.886-1.133]	0.975	0.971[0.895-1.054]	0.486
FT3 (pg/mL)	0.248[0.063-0.984]	0.047^a^	1.323[0.651-2.690]	0.439				
P+A treatment	0.548[0.204-1.468]	0.232	0.748[0.404-1.382]	0.353	0.603[0.229-1.587]	0.306	0.839[0.443-1.586]	0.588

*P* < 0.05 was considered statistically significant. ^a^ represents a statistically significant difference between the two groups

*FT3 *free triiodothyronine, *P *prednisone, *A *aspirin

### Recurrent pregnancy loss and IVF outcome

Baseline demographics and clinical characteristics were comparable between groups (Supplemental Table SII). Based on our analysis, no association was observed between P + A treatment and subsequent pregnancy outcomes in women suffering from recurrent pregnancy loss who had autoimmune thyroid disease (Supplemental Table SIII). However, the small sample size in this study did not provide the adequate power required to evaluate this outcome. Thus, future investigations, preferably studies focusing on randomized controlled trials (RCT), are urgently needed to assess the value of additional treatment in recurrent miscarriage women with TAI.

### Comparison of IVF outcomes of continuous embryo transfers

The comparison of IVF outcomes of continuous embryo transfers in women receiving nothing at the first embryo transfer but obtaining therapy at the subsequent frozen embryo transfers during the same IVF cycle was depicted in Supplemental Table SIV. In other words, this part was a before-after study in the same patients. As shown in the Supplemental Table SIV, the presence of P + A was not beneficial to final reproductive outcomes. There were considerable shortcomings in the limited eligible evidence, the discrepant ratio of cleavage- to blastocyst-stage embryo, and various types of embryos transferred, fresh or frozen-thawed, inevitably reaching a questionable conclusion.

## Discussion

The correlation between antithyroid antibodies, fecundity, and pregnancy outcomes is quite debatable and conflicting. Previously, a meta-analysis of four prospective studies that included 1098 subfertile women undergoing IVF revealed a significant two-fold increase in the risk of the miscarriage of subfertile euthyroid women with TAI compared with a counterpart without TAI [20]. Among those four studies, three studies measured the TPO-Ab and the remaining study measured both TPOAb and TgAb. Of them, one study only recruited participants with unexplained infertility and without a previous history of miscarriages, whereas the other studies included subfertile women irrespective of the cause of infertility or a previous history of miscarriages. Under the circumstances of different ART/IVF protocols, dissimilar underlying etiologies contributing to infertility, and changeable cut-off values for euthyroidism and subclinical hypothyroidism, the 2017 American Thyroid Association pregnancy guidelines was unable to reach a definite conclusion on the link between TAI and ART outcomes. Moreover, levothyroxine treatment was recommended for subclinical hypothyroidism, defined as a TSH >2.5 mIU/L, and considered for euthyroid infertile women with TAI when they attempted to conceive by ART after weighing the pros and cons of levothyroxine supplement [21].

In the past few years following the publication of the 2017 guidelines, two large RCTs assessing the value of levothyroxine on pregnancy outcomes in euthyroid TPO-Ab positive women reported that the use of this drug did not significantly improve miscarriage rate and live birth rate [22, 23]. Despite several limitations (mainly involving fixed levothyroxine doses, undetermined TSH values during early pregnancy following medicine supplement, uncertain population compliance, and the exclusion of women with recurrent miscarriages or positive for other autoimmune antibodies), the large-sample RCT results were essential to specifically evaluate levothyroxine effectiveness in euthyroid women with TAI [24]. Furthermore, a recent meta-analysis including six RCTs demonstrated that levothyroxine could not improve clinical pregnancy outcomes among women who were positive for TPOAb. Indeed, of the meta-analyses that were based on high- to moderate-quality evidence, two trials involved ART, two studies used fixed levothyroxine doses and one investigation enrolled euthyroid or subclinical women [25]. Thus, additional large-scale high-quality research on this particular population is still urgently needed.

Based on the decreased effectiveness of levothyroxine and generalized autoimmune imbalance resulting from thyroid autoimmunity, the impact of P + A treatment on euthyroid women with TAI undergoing their first IVF/ICSI procedure was retrospectively explored. A dynamic and responsive immune system is critical for a successful pregnancy—the first trimester begins in a pro-inflammatory stage that allows implantation and placentation, then it shifts to an anti-inflammatory environment, pivotal for fetal growth, and finally returns to a pro-inflammatory stage suitable for labor and delivery [26]. The pro-inflammatory process initiated during embryo implantation and trophoblast invasion better promote cell clearance, angiogenesis, cell growth, and tolerance, as it is characterized by the presence of angiogenic, growth, and survival factors, as well as cytokines and chemokines [26]. Following implantation, the female immune system induces tolerance towards the embryo, whereas tolerance induction is incomplete in a hyperactive immune system. Subfertile women with autoimmune thyroid disease usually express increased levels of IFNγ from pro-inflammatory Th1 immune cells, along with lower IL-4 and IL-10 from Th2 immune cells compared with control patients without antithyroid antibodies. This suggests that excessively activated pro-inflammatory Th1 cells hamper the onset of a successful pregnancy [27]. Moreover, pinopodes, the spherical protrusions of the epithelial plasma membrane into the lumen, are characterized as classic morphological biomarkers of receptive endometrium favoring implantation [27]. Recently, a euthyroid Hashimoto’s thyroiditis mice model was established to explore the correlation between Hashimoto’s thyroiditis and endometrial receptivity defects. The resulting evidence indicated that Hashimoto’s thyroiditis alone inhibited luminal epithelium development, retarded the formation and development of pinopodes, and decreased expression of receptivity markers, thereby inducing a nonreceptive endometrial milieu and leading to implantation failure [28]. Prednisone, a type of glucocorticoid, is readily absorbed from the gastrointestinal tract and used primarily for its anti-inflammatory effects in many disorders [29]. Several trials revealed that low doses of corticosteroids (10 mg/day) improved IVF pregnancy outcomes in women experiencing immunological infertility and recurrent miscarriages, even in patients with a prior history of 19 consecutive miscarriages [16, 17, 30, 31]. Furthermore, by exposing cleavage-stage mouse embryos to 3 and 30 µM concentrations of prednisolone *in vitro* to assess the embryonic response to direct prednisolone exposure, a recent animal study revealed that exposure to 30 µM prednisolone delayed the embryonic progression, decreased hatching potential, and increased apoptosis in blastocysts. However, 3 µM prednisolone increased proliferation of the inner cell mass, which was incorporated to predict the implantation potential [32]. It is worth mentioning that 3 µM is close to the therapeutic dose and 30 µM is ten-fold higher than the initial level. Experimental evidence in animal models demonstrated that glucocorticoids at higher concentrations could negatively affect oocyte maturation and early embryogenesis. The therapeutic dose of prednisolone reduced post-implantation demise, possibly due to its effects on choriocarcinoma cell lines. Similarly, the latest trial that investigated the role of prednisolone on decidualization and decidual-trophoblast interactions reported that this treatment enhanced trophoblast outgrowth, elevated trophoblast mRNA expression of cell motility gene PLCG1, and altered decidual-trophoblast interactions, yet the clinical consequences of these changes were unknown [33]. Thus, there remains a great need for further research on this topic.

Simultaneously, a low dose of aspirin plays an essential role in improving uterine and ovarian blood flow, enhancing embryo implantation, and sustaining early pregnancy. This stems from its capacity to decrease blood viscosity and increase blood flow, which is secondary to the inhibition of cyclooxygenase-1 and the decreased production of thromboxane-2. In addition, daily low-dose aspirin use is considered safe as it does not affect the menstrual cycle, follicular phase, luteal phase length, or hormone levels across the menstrual cycle [34]. Adjuvant treatment of P + A is recommended to patients with autoantibodies undergoing IVF and its benefits have been demonstrated in several studies [16–18]. However, these trials were published long ago and do not demonstrate the efficacy of this approach in infertile women who were positive only for antithyroid antibodies.

In our study, patients were classified according to their age in three categories: <31 years, 31–37 years, and >37 years, based on our understanding of natural fertility, since its decline begins at 31 years and 37 years old has been recorded as the pivotal age for success rates in treatment programs [35, 36]. Notably, the distribution among age groups was comparable and thus reduced the potential confounding risk of age, as an advanced age increases the chance of de novo chromosomal aberrations in oocytes and, in turn, in the embryo [11, 37, 38]. As for ovarian reserve, age, AMH, AFC, and FSH were all comparable between treated and untreated patients for both fresh and frozen embryo transfer cycles. In addition, couples with significant parental chromosome abnormality, severe oligoasthenospermia, and azoospermatism were excluded from our study as the rate of chromosomal anomaly was 0.24% in the normal semen group, 4.7% in the moderate-to-severe oligoasthenospermia group, and 9.59% in the azoospermia group [39]. A total of 30-50% of implantation failures can be attributed to poor embryo quality and embryo quality was determined by several parameters, primarily the women’s age, ovarian reserve, underlying causes of infertility, and sperm quality. Furthermore, decreased endometrial receptivity was thought to account for two-thirds of these failures [5]. Typically, the endometrium is a direct or indirect target of antithyroid antibodies, prednisone, and aspirin.

Because the harm caused by a single antibody and combined antibodies is not clear, the proportion of positive isolated TPOAb, positive isolated TgAb, and double-positive TPOAb and TgAb were recorded and analyzed in our study, and no significant differences were observed. Moreover, in our study, euthyroidism was defined by a TSH reference value range of 0.35–4.0 mIU/L and the value was comparable between the two groups. The threshold between euthyroid and subclinical hypothyroidism changes over time. Nowadays, the association between elevated maternal TSH concentrations and pregnancy-specific complications appears to be more pronounced when adopting the cut-off point of 4.0 mIU/L, or a population-based reference value than a level of 2.5 mIU/L [40]. Newer guidelines suggested that an upper limit of 4.0 mIU/L should be considered diagnostic compared with the previous guideline of 2.5 mIU/L [21]. Based on the TSH threshold of 4.0mIU/L and recommendations of the 2017 American Thyroid Association, levothyroxine supplementation was not included in our study.

Interestingly, no association between P + A treatment and reproductive outcomes was observed including clinical pregnancy rate, miscarriage rate, and the live birth rate at the first embryo transfer regardless of embryo type (fresh or frozen). Assessed infertile women exhibited normal thyroid tests and no autoimmune antibodies except anti-thyroid antibodies. These finding have not been replicated in other studies and should be interpreted with caution. In 2009, Revelli et al. performed a retrospective study of 329 euthyroid women who were positive for TPOAb, TgAb, or both. The medication prescribed was prednisolone (10 mg/d) and aspirin (100 mg/d), from the day of stimulation to 10 weeks of gestational age and, during that period, P was increased to 30 mg/d for 5 days starting from the day of embryo transfer. This approach was deemed beneficial to pregnancy and implantation rates in contrast with untreated ATA+ patients [18]. In our study, treatment started on the day of embryo transfer and lasted for 2–6 weeks, mainly focusing on improving the micro-immune environment of the implantation site. In a prospective case-control study including 233 consecutive patients, dexamethasone (0.5 mg/d) and acetylsalicylic acid (100 mg/d) starting from the day of embryo transfer and until the end of the 12th week of gestation increased the pregnancy rate and implantation rate when compared with the control group [19]. The inclusion criteria of this prospective study included inherited and acquired thrombophilia, compound heterozygous polymorphisms, and positive anti-nuclear and anti-thyroglobulin antibodies, which were strong indications for steroid hormone and anticoagulant drugs [19]. Coincidentally, the inclusion criteria coincided with the exclusion criteria in our study, which largely explained the conflicting results. Thus, the effective value of treatment maybe not be evident in women with unaffected thyroid function and only thyroid antibodies when compared with those with multiple types of autoantibodies or a history of recurrent pregnancy loss.

In terms of the mixed correlation between TAI and infertility, a recent review published in 2020 contributed to a better understanding of its relevance. By summarizing and analyzing the latest studies since the 2017 guidelines, this review documented that anti-TPO antibodies were associated with infertility in subsets of women, mainly in those with unexplained infertility or polycystic ovarian syndrome (PCOS), but not in all women [24]. Such a conclusion was primarily dependent on a secondary analysis of data from two multicenter RCTs involving 1468 euthyroid infertile women either with unexplained infertility or PCOS [41]. The weak correlation between TAI and IVF reproductive outcomes of the general infertile population partially explained the negative results of our study. Furthermore, a 2020 meta-analysis of 17 studies pinpointed a statistically significant association between recurrent miscarriage and TAI (odds ratio 1.94; 95% CI, 1.43–2.64). The statistical significance and magnitude of the results remained unchanged following sensitivity analyses [42], similarly to the findings in our previous work [43]. Up to now, for euthyroid infertile women with unexplained infertility, PCOS, or recurrent pregnancy loss, little evidence exists concerning the effect of replacement therapy of P + A. In our study, due to the scarcity of a great number of subjects, stratified research does not achieve valid results at the subgroup level. However, based on the fact that P +A treatment improved adverse IVF reproductive outcomes in women with positive antinuclear antibodies [44], unexplained recurrent pregnancy loss [45, 46], repeated implantation failure [45, 47–49], and other immune-related antibodies [15–17, 46, 50, 51], combined treatment is likely to benefit euthyroid infertile women with TAI and unexplained infertility or recurrent pregnancy loss. However, prospective large-sample trials are still required to justify its potency.

Additionally, in our study, it was observed that regardless of the embryo type being transferred, the incidence of abortion was higher but not significant in the treated group than in control patients. As illustrated above, the value of FT3 in the P + A treated group was 2.90 ± 0.39 pg/mL, significantly lower than that of thyroid antibodies-positive untreated subjects (*P* = 0.017). Multivariable logistic regression demonstrated the negative role of FT3 in fetal loss incidence at the first fresh embryo transfer. This was consistent with a preliminary observational study that reported that low serum FT3 levels compromised the beneficial effect of levothyroxine substitution in women with Hashimoto’s thyroiditis [52]. It is widely acknowledged that thyroid hormone transporters, receptors, and their associated proteins are expressed in the ovary, early embryo, endometrium, uterus, and placenta [7]. Simultaneously, the expression of these proteins in the endometrium is dynamic throughout the various phases of the menstrual cycle [53]. It has been documented that receptive endometrium is accompanied by an increased expression of thyroid hormone receptors in normal women [54], whereas decreased expression of thyroid-related proteins in the uterus was observed on the day of implantation in hypothyroid pregnant rats [55]. By binding to thyroid hormone receptors on the placenta and endometrium, as well as regulating the invasive potential of extravillous trophoblasts, thyroid hormone can affect implantation and early fetal development [7]. Furthermore, an optimal T3 value is crucial for ovulation and folliculogenesis, as T3 in combination with FSH enhances proliferation of granulosa cells and inhibits apoptosis of granulosa cell via the PI3K/Akt pathway [7, 56]. To conclude, the aforementioned evidence seemingly suggests that additional levothyroxine should be supplemented in euthyroid infertile women with low but yet normal values of FT3. A novel pathogenesis model of the link between thyroid autoimmunity and fertility may offer us a new perspective [24]. During the early stages of autoimmunity, the main detrimental effects comprise the hostile immune environment impacting the ovary, with TPO as the direct antigen. At this stage, the thyroid hormone response is still intact and levothyroxine treatment is inefficient. As thyroid autoimmunity progresses, thyroid response to hCG stimulation is impaired and thus unable to meet the high thyroid hormone demand during pregnancy. In that situation, treatment with thyroid hormone would prove beneficial [24]. Based on this potential model, distinguishing the different stages is key to achieving an efficient treatment regimen.

Although progress has been made in some areas of autoimmune disorders, little is known about the underlying mechanisms of autoimmune antibodies on reproductive outcome, which represents a challenge for effective treatment research. Organs-on-a-chip, advanced *in vitro* models of multicellular tissue complexes or functional organ units, may help illuminate this intricate connection. Exploiting organ-on-a-chip approaches to model decidualization, implantation, and placentation would enable an in-depth study of the invasive and remodeling behavior of extravillous trophoblast cells, and of uteroplacental circulation that provides vascular supply to the growing fetus [57]. Furthermore, the interaction between antibodies and endometrium and variable expression of immunological factors, as well as glucocorticoid targets, all should be explored.

Our study has several advantages. Firstly, by establishing strict inclusion and exclusion criteria, the possible confounding factors of other autoantibodies and severe detrimental elements of spontaneous miscarriages were controlled for, which allowed us to minimize any patient-related variation and concentrate solely on P + A effects on isolated euthyroid infertile women with TAI. Secondly, only first-time ART users were included and analyzed to investigate a homogeneous, good-prognosis population and provide relevant suggestions for targeted subjects. However, our study also had some limitations. Firstly, it was inevitably limited by its retrospective nature. Secondly, given the variation in TPOAb and TH concentrations in the context of pregnancy, the measurement of longitudinal thyroid parameters during pregnancy was reasonable and necessary, but the changes were not recorded [58]. While the P + A supplement did not improve the live birth rate or pregnancy rate in euthyroid TAI women at the first embryo transfer, the potential benefits of P + A supplementation during pregnancy cannot be ruled out. Additional RCTs are required to determine whether P + A would yield different results on women who test positive for antithyroid antibodies with recurrent implantation failure or with unexplained infertility. Similarly, a higher-risk population with increased recurrent pregnancy loss might be affected differently.

## Conclusions

To conclude, according to the reality of routine thyroid screening, many euthyroid women who test positive for antithyroid antibodies were discovered. Among them, patients who underwent the first IVF cycle without a history of recurrent miscarriages or unexplained infertility were not recommended for the combined treatment of prednisone and aspirin.

## Supplementary Information

**Additional file 1.**

## Data Availability

All data generated or analyzed in this study are included in this manuscript and its supplementary information files.

## Abbreviations

TAI: Thyroid autoimmunity
TPOAb: Thyroperoxidase antibody
TgAb: Thyroglobulin antibody
IVF-ET: *in vitro* fertilization and embryo transfer
FT3: Free triiodothyronine
P + A: Prednisone and aspirin
ART: Assisted reproductive technology
TSH: Thyroid-stimulating hormone
FSH: Follicle-stimulating hormone
AFC: Antral follicle count
SLE: Systemic lupus erythematosus
APS: Antiphospholipid syndrome
DM: Diabetes mellitus
ECL: Electrochemiluminescence
FT4: Free thyroxine
rFSH: Recombinant follicle-stimulating hormone
HMG: Human menopausal gonadotropin
GnRH: Gonadotropin-releasing hormone
hCG: Human chorionic gonadotropin
BMI: Body mass index
AMH: Anti-Mullerian hormone
AFC: Antral follicle count
Gn: Gonadotropin
SPSS: Statistics Package for Social Sciences
RCT: Randomized controlled trial
PCOS: Polycystic ovarian syndrome
