# Supplementary material for: IVF/ICSI outcomes of euthyroid infertile women with thyroid autoimmunity: does treatment with aspirin plus prednisone matter?
Source: BMC Pregnancy Childbirth. 2022 Mar 29;22:263. doi: 10.1186/s12884-022-04532-2 (PMC8966173; doi:10.1186/s12884-022-04532-2)
Supplement: Supplementary file 1 — Additional file 1. [file 12884_2022_4532_MOESM1_ESM.docx]

Supplement Table SI. Causes of infertility

| Cycles | Fresh embryo transfer cycles | | | Frozen embryo transfer cycles | | |
| --- | --- | --- | --- | --- | --- | --- |
|  | Control group n=113 | P+A group n=74 | P value | Control group n=83 | P+A group n=76 | P value |
| Cause of infertility |  |  |  |  |  |  |
| Male factors, n (%) | 38.9 (44/113) | 37.8 (28/74) | 0.880 | 36.1 (30/83) | 34.2 (26/76) | 0.799 |
| Tubal factors, n (%) | 64.6 (73/113) | 62.2 (46/74) | 0.735 | 55.4 (46/83) | 56.6 (43/76) | 0.883 |
| Ovarian dysfunction, n (%) | 15.9 (18/113) | 13.5 (10/74) | 0.651 | 34.9 (29/83) | 26.3 (20/76) | 0.239 |
| Endometriosis, n (%) | 12.4 (14/113) | 4.1 (3/74) | 0.053 | 9.6 (8/83) | 9.2 (7/76) | 0.927 |
| Uterus factors, n (%) | 8.8 (10/113) | 4.1 (3/74) | 0.207 | 12.0 (10/83) | 3.9 (3/76) | 0.063 |
| Idiopathic, n (%) | 4.4 (5/113) | 5.4 (4/74) | 0.759 | 7.2 (6/83) | 9.2 (7/76) | 0.649 |

Supplemental Table SII Baseline demographics and clinical characteristics in women with recurrent pregnancy loss

| Cycles | Fresh embryo transfer cycles | | | Frozen-thawed embryo transfer cycles | | |
| --- | --- | --- | --- | --- | --- | --- |
|  | Control group n=21 | P+A group n=15 | P value | Control group n=7 | P+A group n=10 | P value |
| Age (yrs) | 30.90±3.22 | 32.80±3.10 | 0.086 | 30.43±5.86 | 32.00±4.69 | 0.548 |
| BMI (kg/m^2^) | 22.76±2.89 | 23.17±3.52 | 0.699 | 23.40±3.23 | 21.19±1.79 | 0.089 |
| Previous miscarriages | 2.0 (2.0-3.0) | 2.0 (2.0-2.0) | 0.657 | 2.0 (2.0-3.0) | 2.0 (2.0-3.0) | 1.000 |
| Duration of infertility (yrs) | 2.0 (1.0-3.5) | 2.0 (1.0-5.0) | 0.547 | 2.0 (1.0-2.0) | 2.0 (1.0-4.0) | 0.417 |
| TSH (mIU/L) | 1.94±1.01 | 2.30±0.98 | 0.289 | 1.5 (1.0-1.7) | 2.1 (0.7-3.2) | 0.740 |
| FT4 (ng/dL) | 1.04±0.17 | 1.01±0.09 | 0.529 | 0.99±0.15 | 1.04±0.07 | 0.336 |
| FT3(pg/mL) | 3.01±0.43 | 2.77±0.37 | 0.086 | 3.10±0.44 | 3.00±0.37 | 0.645 |
| Basal FSH(IU/L) | 5.77±1.23 | 5.22±0.87 | 0.146 | 5.57±2.26 | 5.73±1.81 | 0.874 |
| AMH (ng/mL) | 2.5 (1.6-3.7) | 3.0 (2.0-3.6) | 0.612 | 4.4 (3.1-5.1) | 2.5 (1.5-6.6) | 0.193 |
| AFC | 12.5 (7.8-14.3) | 10 (9.5-21.5) | 0.679 | 12.0 (9.0-17.0) | 14.0 (8.0-16.0) | 0.905 |

Supplemental Table SIII IVF outcomes in women with recurrent pregnancy loss

| Cycles | Fresh embryo transfer cycles | | | | Frozen-thawed embryo transfer cycles | | | |
| --- | --- | --- | --- | --- | --- | --- | --- | --- |
|  | Control group n=21 | P+A group n=15 | F value | P value | Control group n=7 | P+A group n=10 | F value | P value |
| Total Gn Dose (IU) | 2007.14±672.35 | 2297.50±595.05 |  | 0.190 | 2175.00±950.66 | 1788.75±735.28 |  | 0.359 |
| Stimulation length (d) | 10.0 (8.5-11.0) | 10.0 (10.0-12.0) |  | 0.309 | 9.57±1.51 | 8.90±2.08 |  | 0.478 |
| Number of oocytes retrieved | 9.52±4.30 | 10.93±5.64 |  | 0.400 | 15.86±1.35 | 9.70±6.43 |  | 0.055 |
| Gn dose/oocyte (IU) | 225.0 (133.1-366.2) | 208.9 (144.2-375.0) |  | 0.950 | 126.6 (88.2-208.9) | 122.3 (90.0-646.9) |  | 0.601 |
| Fertilization rate (%) | 91.0 (182/200) | 89.6 (147/164) | 0.193 | 0.660 | 78.4 (87/111) | 81.4 (79/97) | 0.302 | 0.583 |
| Cleavage rate (%) | 91.8 (167/182) | 95.2 (140/147) | 1.578 | 0.209 | 92.0 (80/87) | 96.2 (76/79) | 1.320 | 0.251 |
| Available embryo rate (%) | 49.7 (83/167) | 49.3 (69/140) | 0.005 | 0.942 | 61.3 (49/80) | 60.5 (46/76) | 0.009 | 0.926 |
| Implantation rate (%) | 24.4 (10/41) | 34.5 (10/29) | 0.848 | 0.357 | 45.0 (5/11) | 38.9 (7/18) |  | 1.000 |
| Pregnancy rate (%) | 61.9 (13/21) | 53.3 (8/15) |  | 0.736 | 71.4 (5/7) | 50.0 (5/10) |  | 0.622 |
| Clinical pregnancy rate (%) | 47.6 (10/21) | 53.3 (8/15) |  | 1.000 | 71.4 (5/7) | 50.0 (5/10) |  | 0.622 |
| miscarriage rate (%) | 10.0 (1/10) | 25.0 (2/8) |  | 0.559 | 20.0 (1/5) | 60.0 (3/5) |  | 0.524 |
| Live birth rate (%) | 42.9 (9/21) | 40.0 (6/15) |  | 1.000 | 57.1 (4/7) | 20.0 (2/10) |  | 0.162 |

Supplemental Table SIV Comparison of IVF outcomes of continuous embryo transfers in patients with medication

| Cycles | Fresh embryo transfer cycles | | | | Frozen-thawed embryo transfer cycles | | | |
| --- | --- | --- | --- | --- | --- | --- | --- | --- |
|  | Former fresh embryo transfer n=13 | Treatment frozen embryo transfer n=13 | F value | P value | Former frozen embryo transfer n=3 | Treatment frozen embryo transfer n=3 | F value | P value |
| Embryo stage, n (%) |  |  |  |  |  |  |  |  |
| Cleavage stage | 13 (100.0%) | 5 (45.5%) |  | 0.003* | 3 (100.0%) | 0 (0.0%) |  | 0.100 |
| Blastocyst stage | 0 (0.0%) | 6 (54.5%) |  |  | 0 (0.0%) | 3 (100.0%) |  |  |
| Number of embryos transferred | 2.00±0.00 | 1.77±0.44 |  | 0.336 | 2.00±0.00 | 1.33±0.58 |  | 0.200 |
| Implantation rate (%) | 3.8 (1/26) | 26.1 (6/23) | 3.281 | 0.070 | 0.0 (0/6) | 25.0 (1/4) |  | 0.400 |
| Pregnancy rate (%) | 7.7 (1/13) | 46.2 (6/13) |  | 0.073 | 0.0 (0/3) | 100.0 (3/3) |  | 0.464 |
| Clinical pregnancy rate (%) | 7.7 (1/13) | 38.5 (5/13) |  | 0.160 | 0.0 (0/3) | 33.3 (1/3) |  | 1.000 |
| miscarriage rate (%) | 100.0 (1/1) | 40.0 (2/5) |  | 1.000 | 0.0 (0/0) | 100.0 (1/1) |  | / |
| Live birth rate (%) | 0.0 (0/13) | 23.1 (3/13) |  | 0.220 | 0.0 (0/3) | 0.0 (0/3) |  | / |
